# Concurrent Validity of the Operationalization of High-Impact Pain Construct in the Health and Retirement Study

**DOI:** 10.21203/rs.3.rs-6733184/v1

**Published:** 2025-05-28

**Authors:** Javier A. Tamargo, Yenisel Cruz-Almeida

**Affiliations:** University of Florida; University of Florida

**Keywords:** Pain, Geroscience, Epidemiology

## Abstract

**Purpose:**

Chronic pain epidemiology is hindered by inconsistent definitions and methods. The U.S. National Pain Strategy introduced high-impact chronic pain as a pain surveillance standard, defined as chronic pain that interferes with daily life. This study aimed to validate the operationalization of high-impact pain in the Health and Retirement Study (HRS), a large, nationally representative cohort of older adults.

**Patients and methods:**

We analyzed data from the 2010 HRS pain module. High-impact pain was operationalized with two questions that have been fielded in the HRS nearly since its inception. Pain intensity and pain-related disability were assessed using numeric rating scales and the Pain Disability Index (PDI). We used Wilcoxon rank-sum tests and logistic regressions to compare high- versus low-impact pain. Pain impact was also assessed at a 2-year follow-up in 2012.

**Results:**

Out of 508 participants, 335 (65.9%) reported high-impact pain. Those with high-impact pain had significantly higher pain-related disability (median PDI: 48 [33, 60] vs. 19 [8, 36], P<0.0001), average pain intensity (median: 6 [4, 8] vs. 5 [3, 6], P<0.0001) and were more likely to report chronic (OR: 1.75 [95% CI: 0.19, 2.58]) and constant (OR: 3.09 [1.93, 4.93]) pain. High-impact pain was associated with a relative risk of 1.80 (1.53, 2.11) for continued high-impact pain at a 2-year follow-up.

**Conclusion:**

The HRS operationalization of high-impact pain demonstrates strong concurrent validity with established measures of pain disability, intensity, and impact. The HRS provides a valuable tool for advancing pain research, particularly in aging populations.

## Introduction

Historically, surveillance of chronic pain has faced several limitations, such as a lack of standardized methods and definitions, hindering the accurate monitoring of its prevalence and impact. To that end, in 2016, the U.S. National Pain Strategy (NPS) Population Research Workgroup proposed high-impact chronic pain (HICP) as a pain surveillance standard, since the term recognizes both the duration (i.e., 3 months or longer) and its interference with work or life activities.^1^ The NPS defined HICP as chronic pain that is “associated with substantial restriction of participation in work, social, and self-care activities.” Yet, in a review of chronic pain surveillance systems in the U.S., Duca et al. found a lack of comprehensive and consistent data collection methods, particularly for HICP. These gaps have impeded the ability to fully understand and address the burden of HICP, highlighting the need for improved and standardized surveillance measures.^2^

The Health and Retirement Study (HRS) is a longstanding longitudinal panel study conducted by the University of Michigan and supported by the National Institute on Aging. Since its inception in 1992, the HRS has surveyed a representative sample of over 20,000 Americans aged 50 and older every two years. As such, the HRS offers a rich source for aging studies, but it has been underutilized for pain research. Recently, we used HRS data to evaluate the relationship between high-impact pain and epigenetic aging, corroborating and expanding our previous findings in a nationally representative sample of middle-aged and older adults in the U.S.^3^ High-impact pain was measured using a series of questions that have been fielded in nearly every HRS core interview since its inception: (1) “Are you often troubled with pain?,” (2) “How bad is the pain most of the time: mild, moderate, or severe?,” and (3) “Does the pain make it difficult for you to do your usual activities such as household chores or work?” Participants who reported being often troubled by pain and indicated that it interfered with their usual activities were classified as having high-impact pain. Recognizing that duration was not explicit, we did not refer to high-impact pain as chronic. To validate that the operationalization of high-impact pain was consistent with its construct, we conducted several analyses with objective and subjective measures of physical function. Our findings showed that high-impact pain was associated with significantly greater functional limitations, including poorer performance in physical function tests (i.e., grip strength, balance, and gait speed) and higher self-reported difficulties in mobility, large muscle tasks, activities of daily living (ADLs), instrumental ADLs, gross motor skills, and fine motor skills. Additionally, individuals with high-impact pain were more likely to report moderate or severe pain compared to those with low-impact pain, indicating a higher pain burden.

Building on those findings, we aimed to further validate the operationalization of high-impact pain in the HRS using a pain module that was exclusively included in the 2010 wave. By leveraging these data, we examined the concurrent validity, consistency, and robustness of our previous operationalization and explored its applicability for future pain research. We hypothesized that high-impact pain, as operationalized in the HRS, would be associated with greater pain-related disability and impact, as well as pain intensity.

## Material and methods

For this analysis, we used data from the 2010 HRS pain module. In the HRS, optional experimental modules are administered at the end of the core interview and are designed to be brief explorations of new topics or to enhance the information already collected. Generally, each module is completed by a 10% random sample of the core. The pain module included a series of detailed questions designed to capture various aspects of pain, including its duration, intensity, causes, and impact. Pain intensity on average, at its worst, and at its least was assessed with a numeric rating scale (NRS) ranging from 0 (“no pain”) to 10 (“pain as bad as you can imagine”). The module also included the Pain Disability Index (PDI), a validated instrument that evaluates how pain interferes with seven key areas of life: family/home responsibilities, recreation, social activities, work, sexual behavior, self-care, and essential life-support activities. Each area is rated on a scale from 0 (no disability) to 10 (total disability), with the total score ranging from 0 to 70. Higher scores indicate greater disability and impact on life activities due to pain.^4^ The full module can be seen in HRS documentation.^5^

Pain impact classification was determined using the 2010 core interview questions, as previously mentioned.^3^ We also examined pain impact longitudinally, using the 2012 core interview questions. This study was deemed exempt from Institutional Review Board review because it utilized publicly available de-identified data.

### Statistical analysis

Group differences in pain-related disability and pain intensity (continuous outcomes) were evaluated with Wilcoxon rank-sum tests and reported as median (interquartile range [IQR]). We also calculated the point-biserial correlation coefficients (*r*_pb_) as a measure of effect size. The point-biserial correlation quantifies the strength and direction of the association between a dichotomous variable and a continuous variable. Differences in categorical outcomes were evaluated with logistic regressions and reported as counts and percentages with odds ratios (OR) plus 95% confidence intervals. To evaluate pain impact reports longitudinally, we performed a generalized linear regression accounting for repeated measures and calculated the relative risk plus 95% confidence interval of high-impact pain at follow-up. Results were considered statistically significant at α = 0.05. The data analysis for this paper was generated using SAS software, version 9.4. Copyright © 2020 SAS Institute Inc. SAS and all other SAS Institute Inc. product or service names are registered trademarks or trademarks of SAS Institute Inc., Cary, NC, USA.

## Results

Out of 1,926 respondents who opted into the module, 778 reported experiencing pain that lasted for 1 week or longer in the past year; thus, they were prompted to complete the module. We further excluded those who did not report being often troubled with pain (n = 270). The final analytic sample for this report was 508 participants, with 335 (65.9%) of those who reported high-impact pain. Sample characteristics are shown in Table 1.

### Cause of pain

Respondents were asked about the main cause of their worst pain episode in the last year. The most common reason was “illness, organic or degenerative disorder (e.g. arthritis, osteoporosis)” reported by 47.8% of participants, followed by “accident or injury” (20.7%), and “other” (17.9%). There was no significant difference in the main cause of pain reported by people with high-impact pain and low-impact pain (P = 0.69).

### Pain intensity

Individuals with high-impact pain, compared to those with low-impact pain, reported higher pain intensity on average, at its worst, and at its least painful on a numeric rating scale (NRS) ranging from 0 (“no pain”) to 10 (“pain as bad as you can imagine”).

### Pain-related disability

Similarly, individuals with high-impact pain reported greater pain-related disability than those with low-impact pain on all PDI items, with median (interquartile range) PDI scores of 48 (33, 60) and 19 (8, 36), respectively (P < 0.0001). Notably, the differences between groups were more pronounced for pain-related disability (*r*_pb_ PDI: 0.451) than for pain intensity (*r*_pb_ average pain: 0.247).

### Duration of pain

Respondents were asked if their most severe pain episode lasted less than 1 month, 2 to 3 months, 4 to 6 months, 7 months to 1 year, or more than 1 year. Since the National Center for Health Statistics defines chronic pain as pain experienced on “most days or all days in the past 3 months” ^6^, we categorized responses of “2 to 3 months” or higher as chronic pain. Individuals with high-impact pain, compared to those with low-impact pain, were more likely to report chronic pain (72.3% vs. 59.9%, respectively; P = 0.005), including pain lasting over 1 year (41.6% vs. 32.6%, respectively; P = 0.049). We also examined pain reports at the subsequent 2012 wave among the subset of participants with valid pain impact data at follow-up (n = 460). Among individuals with high-impact pain in 2010, the majority continued to report high-impact pain in 2012 (69.4%), while 12.3% and 18.4% reported low-impact pain or no pain, respectively. In the subset of participants who reported high-impact pain lasting less than 1 month in 2010, 69.5% continued to report high-impact pain in 2012. In contrast, among those who reported low-impact pain in 2010, 38.0%, 34.0%, and 28.0% reported high-impact pain, low-impact pain, and no pain in 2012, respectively. Individuals with high-impact pain at baseline had a relative risk of 1.80 (95% CI: 1.53, 2.11; P < 0.0001) for high-impact pain at follow-up compared to those with low-impact pain at baseline.

### Frequency of pain

Additionally, individuals with high-impact pain were more likely to report constant pain, but not flare-ups.

### Pain impact

When prompted to think of their worst episode of pain, individuals with high-impact pain were more likely to report that pain interfered with paying for needs, going to the emergency room because of pain, and taking prescription pain medications (Table 3).

## Discussion

Our analysis demonstrated concurrent validity of the operationalization of high-impact pain in the HRS with two simple questions that have been fielded since its inception. Individuals who report high-impact pain, compared to those who report low-impact pain, also report greater pain-related disability, pain interference in paying for needs, going to the emergency room due to pain, and using prescription medications due to pain. These findings support and enhance our previous observations regarding the operationalization of high-impact pain in the HRS, including significantly greater functional limitations in both self-reported and objective measures of physical function.^3^ Additionally, individuals with high-impact pain reported significantly higher pain intensity, which is also consistent with our previous finding.^3^ It should be noted, however, that differences between pain impact groups were markedly greater in measures of disability and interference than for pain intensity. This suggests that high-impact pain, as measured in the HRS, demonstrates some level of discriminant validity by showing greater sensitivity to pain interference rather than intensity. This is expected by the current idea that high-impact pain is not solely driven by its intensity but mostly by its negative impact on daily function.^1,7^

Although the temporality of pain is not explicitly acknowledged in the HRS core questions, we posit that the measurement is highly relevant to HICP. Indeed, about 70% of participants with high-impact pain can be classified as having HICP. Moreover, nearly 70% of those with sub-chronic high-impact pain at baseline continued to report high-impact pain at a 2-year follow-up. As such, it’s plausible that most cases of sub-chronic high-impact pain will eventually become chronic as well.

Overall, our findings indicate that the operationalization of high-impact pain in the HRS is a valid and reliable measure, consistent with the definition proposed by the U.S. National Pain Strategy.^8^ As such, the rich and diverse data source offered by the HRS offers several advantages for advancing pain and aging research.

## Conclusion

The Health and Retirement Study offers a reliable measure of high-impact pain that demonstrates concurrent validity with measures of pain-related disability and interference, as well as pain intensity and impact. As such, the rich data source provided by the HRS can be leveraged to advance pain research. For epidemiological pain research to move forward, it is critical to identify and implement validated measures that capture the complex, multidimensional nature of pain through brief, low-burden assessments.

## Figures and Tables

**Table 1:** Participant characteristics by pain-impact groups in the Health and Retirement Study

		Low-impact pain N = 173 (34.1)	High-impact pain N = 335 (65.9)	
		N (%) / Mean ± SD	N (%) / Mean ± SD	P-value
Age	Years	67.2 ± 12.1	63.9 ± 11.1	0.003^a^
Race	White/Caucasian	140 (80.9)	239 (71.3)	0.041^b^
	Black/African American	21 (12.1)	70 (80.9)	
	Other	12 (6.9)	26 (7.8)	
Ethnicity	Hispanic	19 (11.0)	38 (11.3)	0.903^b^
Sex	Female	68 (39.31)	120 (35.8)	
Education	Less than high school	23 (13.3)	81 (24.2)	0.013^b^
	High school or GED	63 (36.2)	123 (36.7)	
	Some college	53 (30.6)	87 (26.0)	
	Bachelor’s degree or higher	34 (19.7)	44 (13.1)	
Body mass index	kg/m^2^	29.2 ± 5.5	31.1 ± 7.5	0.001^a^

a Independent samples T-test

b Chi-square test

**Table 2:** Numeric rating scale items in the 2010 Health and Retirement Study (HRS) Pain Module

		Low-Impact Pain		High-Impact Pain			
	Question item	N	Median (IQR)	N	Median (IQR)	*r_pb_* ^a^	P-value^b^
**Pain Intensity**	0 to 10 rating of worst pain	172	8 (6, 9)	332	8 (7, 10)	0.228	<0.0001
	0 to 10 rating of least pain	171	2 (2, 5)	331	4 (2, 6)	0.219	<0.0001
	0 to 10 rating of average pain	172	5 (3, 6)	330	6 (4, 8)	0.247	<0.0001
**Pain Disability Index**	0 to 10 rating of impact on family/home responsibilities	168	4 (2, 8)	332	8 (5, 9)	0.371	<0.0001
	0 to 10 rating of impact on recreation activities	167	5 (2, 9)	332	9 (6, 10)	0.370	<0.0001
	0 to 10 rating of impact on social activities	169	4 (0, 8)	329	8 (5, 10)	0.372	<0.0001
	0 to 10 rating of impact on work	162	3 (0, 8)	328	8 (5, 10)	0.425	<0.0001
	0 to 10 rating of impact on sex life	159	2 (0, 10)	302	9 (3, 10)	0.321	<0.0001
	0 to 10 rating of impact on basic activities of daily living (ADLs)	167	2 (0, 5)	331	6 (3, 8)	0.315	<0.0001
	0 to 10 rating of impact on essential activities (eating, sleeping, breathing)	168	1 (0, 5)	329	5 (0, 8)	0.283	<0.0001
	Pain Disability Index total score (0 to 70)	149	19 (8, 36)	293	48 (33, 60)	0.454	<0.0001

a Point-biserial correlation

b Wilcoxon rank-sum test

**Table 3:** Categorical items in the 2010 Health and Retirement Study (HRS) Pain Module

		Low-Impact Pain N = 173 (34.1)		High-Impact Pain N = 335 (65.9)			
	Question item	n/total	%	n/total	%	OR (95% CI)	P-value
Duration/Frequency	Chronic pain	103/172	59.9	240/332	72.3	1.75 (0.19, 2.58)	0.005
	1 + years	56/172	32.6	138/332	41.6	1.47 (1.01, 2.17)	0.049
	High-impact pain after 2 years	57/150	38.0	215/310	69.4	1.80 (1.53, 2.11)^a^	<0.0001
	Constant pain	123/173	71.1	296/335	88.4	3.09 (1.93, 4.93)	<0.0001
	Flare-ups	249/329	75.4	129/171	75.7	1.01 (0.66, 1.56)	0.952
	Difficulty sleeping due to pain	116/172	67.4	251/334	75.2	1.46 (0.97, 2.19)	0.066
Worst pain episode	Interfered with paying for needs	14/171	8.2	95/334	28.4	4.46 (2.46, 8.09)	<0.0001
	Take over-the-counter meds due to pain	121/171	70.8	233/335	69.6	0.94 (0.63, 1.41)	0.779
	Go to doctor due to pain	125/172	72.7	261/335	77.9	1.33 (0.87, 2.03)	0.190
	Try alternative therapies due to pain	72/172	41.9	131/334	39.2	0.90 (0.62, 1.30)	0.566
	Go to emergency room due to pain	27/172	15.7	101/335	30.2	2.32 (1.45, 3.72)	0.0004
	Take prescription meds due to pain	104/172	60.5	264/335	78.8	2.43 (1.63, 3.64)	<0.0001

a Relative risk (95% confidence interval)
